# A portable device for studying the effects of fluid flow on degradation properties of biomaterials inside cell incubators

**DOI:** 10.1093/rb/rby026

**Published:** 2018-12-24

**Authors:** Wensen Jiang, Jiajia Lin, Alex H Chen, Jianwei Pan, Huinan Liu

**Affiliations:** 1 Department of Bioengineering, Materials Science and Engineering Program, Stem Cell Center; 2 Department of Mechanical Engineering, University of California, Riverside, Riverside, CA, USA; 3 Department of Bioengineering, University of California, Riverside, Riverside, CA, USA; 4 Department of Neurosurgery, The First Affiliated Hospital of Zhejiang University, Hangzhou, China

**Keywords:** portable flow device, impedance-driven pump, biomaterials, body fluids, magnesium (Mg) degradation

## Abstract

A portable device was designed and constructed for studying the properties of biomaterials in physiologically relevant fluids under controllable flow conditions that closely simulate fluid flow inside the body. The device can fit entirely inside a cell incubator; and, thus, it can be used directly under standard cell culture conditions. An impedance-driven pump was built in the sterile flow loop to control the flow rates of fluids, which made the device small and portable for easy deployment in the incubator. To demonstrate the device functions, magnesium (Mg) as a representative biodegradable material was tested in the flow device for immersion degradation under flow versus static conditions, while the flow module was placed inside a standard cell incubator. The flow rate was controlled at 0.17 ± 0.06 ml/s for this study; and, the flow rate is adjustable through the controller module outside of incubators for simulating the flow rates in the ranges of blood flow in human artery (0.05 ∼0.43 ml/s) and vein (0.02 ∼0.08 ml/s). Degradation of Mg under flow versus static conditions was characterized by measuring the changes of sample mass and thickness, and Mg^2+^ ion concentrations in the immersion media. Surface chemistry and morphology of Mg after immersion under flow versus static conditions were compared. The portable impedance-driven flow device is easy to fit inside an incubator and much smaller than a peristaltic pump, providing a valuable solution for studying biomaterials and implants (e.g. vascular or ureteral stents) in body fluids under flow versus static conditions with or without cells.

## Introduction

In recent years, biodegradable materials have attracted significant interests for biomedical applications. Biodegradable materials require additional testing on degradation properties and the effects of degradation on biological systems, when compared with the conventional non-degradable biomaterials [1–4]. For example, the flow conditions of the medium around the materials could greatly influence the behaviors of cells and cell-material interactions due to shear stress [5]. Moreover, the shear stress and the transport of the degradation products could also influence the degradation rates of biodegradable materials [1–4]. Transport of degradation products under flow condition could also change the local concentrations of degradation products, which could further affect cell behaviors [6–10]. To study the effects of fluid flow on the behaviors of biomaterials, cells and the cell-biomaterial interactions, a flow device is frequently used to mimic typical flow conditions in the body such as blood flow and urine flow.

Traditionally, peristaltic pump has been widely used for fluid dispensing for flow devices [4, 11]. However, the peristaltic pump is generally difficult for sterilization and is not suitable to be placed inside cell culture incubators that are maintained at 37°C and are humidified. Peristaltic pumps are usually placed outside of an incubator [11] or inside an incubator with some protection [4]. If the peristaltic pump is placed outside of an incubator, it would be difficult for some *in vitro* studies that prefer the standard culture environment inside incubators. If the peristaltic pump is placed inside an incubator with some protection, adjusting the flow conditions would typically require repeated opening of incubator doors that are undesirable for many *in vitro* studies. The commercially available axial flow pump is also not ideal for the *in vitro* studies that require sterile conditions, because the pump turbine directly contacts the culture media, which makes it difficult for cleaning and sterilization and thus raises the concerns of contamination. It is preferred to have a small-size, portable and easy-to-setup flow device as an alternative that can function as effective as a peristaltic pump and can fit inside an incubator, while the flow rate can be adjusted outside without opening the incubator doors.

In this study, an impedance-driven pump was used to construct the flow device. Impedance pumps generate flow by ‘pressing’ the elastic tube at an asymmetric position. Previous studies [12–14] revealed the potentials of the impedance-based pumps for flow dispensing for medical applications. The impedance pump is affordable, flexible and small enough to fit into any flow loops, making it an ideal pump for building a portable flow device that fits inside an incubator easily. This article reports the design and construction of the flow device driven by an impedance pump, and testing of the flow device using a model biodegradable material; that is, pure magnesium (Mg). Mg is a biodegradable metal that is promising for a broad range of biological applications, such as orthopedic [8–10], cardiovascular [6, 7], neurological [15–17] and urological applications [18, 19]. The flow device constructed in this study can be used for *in vitro* testing of all biodegradable materials such as biodegradable metals, polymers and ceramics under flow conditions, as well as non-degradable biomaterials, if their intended *in vivo* applications involve fluid flow. Moreover, this flow device can be used for a wide range of studies on biomaterials and medical devices in various body fluids (e.g. blood, plasma, serum, urine) under flow conditions, such as cardiovascular stents, neurovascular stents, flow diverter, ureteral stents, etc.

## Materials and methods

### Assembly of the flow device

**Figure 1:**
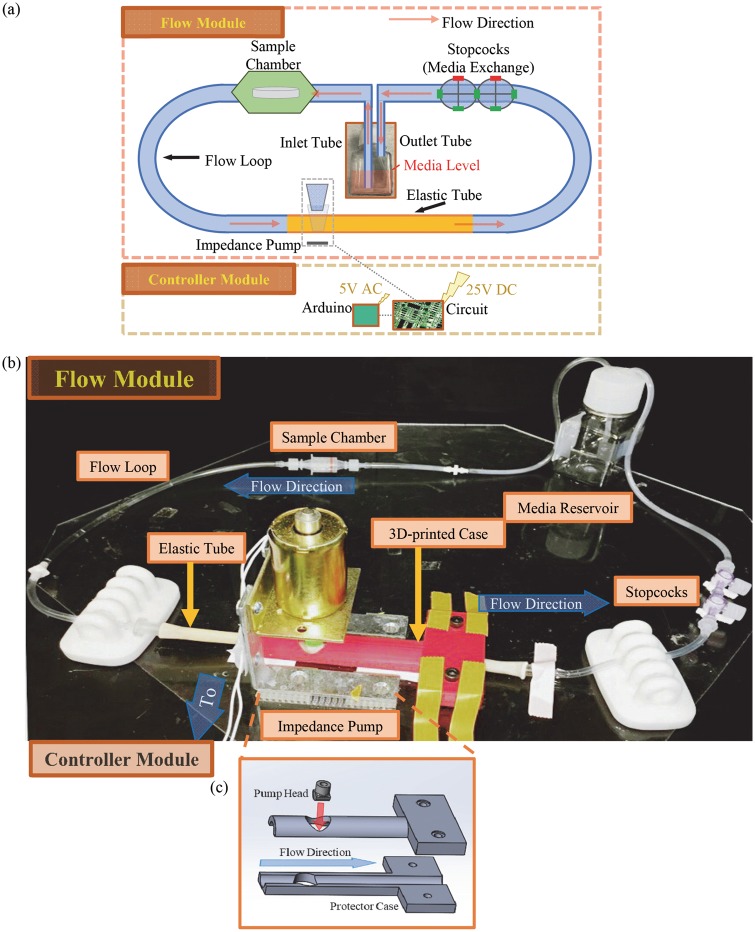
The flow device consists of flow module and controller module. (**a**) The schematic illustration of the flow device. The components are not shown at the ratio of actual size. (**b**) Optical photo of the flow module of the flow device. (**c**) 3D illustrations of protector case and pump head. The respective SolidWorks files used for 3D-printing of pump head and protector case are provided in Supplementary Material. The red arrow and blue arrow in (c) show the direction of pump head movement and flow loop, respectively

The optical photo and schematic illustration of the flow device are shown in Fig. 1. The flow device is composed of a flow module and a controller module. The flow module contains six major components: the impedance pump, the elastic tube, the sample chamber, the media reservoir, the stopcocks and the flow loop. The controller module is the custom-built electronic component that controls the pressing frequency of the impedance pump.

#### Assembly of the flow module

The flow module was secured onto a transparent plastic plate for convenient transportation in and out of a standard cell incubator. The impedance pump provided the driving force for the fluid flow. A solenoid (Guardian Electric, 24 VDC) was mounted to a metallic support, which was previously described [12]. A 3D-printed pump head was mounted onto the solenoid to provide the optimal driving force and to reduce the localized stress on the elastic tube. The elastic tube was made of rubber with an internal diameter (ID) of ¼ inch. A 3D-printed protector case was created to position the pump and the elastic tube. The pump head and the protector case were designed using SolidWorks and then fabricated using a 3D printer (Axiom Dual, Airwolf 3D). The 3D designs of the pump head and protector case are shown in Fig. 1c and the respective files in SolidWorks are provided in Supplementary Material. The press head was placed on top of the elastic tube, and was touching the tube but not pressing the tube when the power was off. The pressing site was 7 cm away from one end of the elastic tube and 15 cm away from the other end of the elastic tube (Fig. 1a), which determined the flow direction. The flow direction was set to be counter clockwise in this study.

A sterilized disposable chamber [P3D-10 chambers, Item # 44-C20.002, HypOxygen] was used as the sample chamber in this study. The sample was placed in the chamber for the immersion test under flow and static conditions. A media bottle from Thermo Fisher Scientific was used as the media reservoir. Two holes were drilled on the cap of media bottle to allow the tube to pass through. The tubes were secured onto the cap of media bottle using a polydimethylsiloxane (PDMS) sealing ring that was cast from PDMS precursors (SORTA-Clear^®^ 18, Smooth-On, Inc.). The PDMS sealing ring isolated the media in the entire flow loop from the outside environment, making it a closed-loop system. The flow tube that connects the reservoir to the sample chamber was immersed below the media as fluid inlet, while the flow tube in the reservoir that connects to the stopcocks was hanging above the media level, that is, not immersed, as outlet.

The stopcocks are composed of two neighboring stopcocks (four-way stopcock, Nordson MEDICAL). The function of the stopcocks is to feed the media to and retrieve the media from the loop. The flow loop is made of a non-elastic plastic tube with an ID of 1/8 inch and 3/16 inch of (TYGON^®^ ND-100-65 Medical/Surgical Plastic Tubing, Clear, purchased from Amazon.com). In addition, fitting components and adapters were used to connect different sections of the flow loop, e.g. connecting the elastic tube of the impedance pump to the flow loop (Catalog # 3060-9, catalog # MTLL230-6005, catalog # CC-1, Nordson MEDICAL).

#### Assembly of the controller module

The controller module controls the pressing motion of the impedance pump, and therefore controls the flow rate of media in the flow loop. The controller module includes an Arduino microcontroller and a transistor circuit (breadboard) that was designed previously [12]. The Arduino microcontroller provided the pulsed signal to the press head. A 5 V AC power supply was applied to the Arduino Uno R3. A 25 V DC power supply was applied to the transistor circuit that drives the motion of press head. In this study, we used the Arduino microcontroller to achieve a 145-µs period of pressing cycle that consisted of a 45-µs pressing (on) and 100-µs releasing (off); that is, a duty cycle of 31.03% and a frequency of 6.90 Hz.

The controller module was placed outside of the incubator because the humidified environment inside the incubator could affect the functionality of electronics and shorten their lifetime. Commercially available standard incubators usually have an outlet to allow the cables to pass through and be easily connected to the outside supplies of power or gas. Placing the controller module outside of incubators also allows the users to adjust flow conditions easily without opening incubators. Repeated opening of incubators is undesirable for cell studies because that could interrupt the culture environment and thus affect the cell behaviors.

### Immersion study for biodegradable Mg under flow and static conditions

A 10 × 10 × 1 mm square-shaped pure Mg (99.9% purity, as-rolled, Alfa Aesar, Ward hill, MA, USA) was polished using up to 800-grit SiC polish paper (Ted Pella Inc. Redding, CA, USA). The sample was then sonicated using a VWR^®^ symphony™ Ultrasonic Cleaners in acetone for 15 min and in ethanol for 15 min to degrease and clean each sample.

The flow module was assembled and connected in a biosafety laminar flow hood following the layout in Fig. 1b. The press head, the protector case and the plastic plate were disinfected by spraying with the 70% ethanol and placed in the biosafety laminar flow hood to dry in air. The rest of parts for the flow module were disinfected by soaking in 100% ethanol for 2 h and then dried in air for 5 h in a biosafety laminar flow hood under a sterile environment. The sample chamber was already in a sterilized package, and no further disinfection was needed. The disinfected Mg sample was placed in the sample chamber under the biological laminar flow hood to keep them sterile. To wash the flow loop, a 50 ml phosphate-buffered saline (PBS) was fed into the flow loop through the stopcocks and then retrieved. After washing, 50 ml media (EGM^TM^-2, No. cc-3162, Lonza) was fed into the flow loop through the stopcocks. EGM^TM^-2 was used in this study because it is the media widely used for culturing human umbilical vein endothelial cells (HUVECs), relevant for vascular applications such as stents and for future studies with HUVECs in the culture [6, 7]. The EGM^TM^-2 media also contains proteins that are important for *in vitro* cell studies and degradation studies, but are absent in the widely used simulated body fluids (SBF). The composition, ionic strength and osmolality of EGM^TM^-2 media were reported previously in comparison with SBF and human blood plasma [7].

The entire assembled flow module (excluding the controller module) was put into an incubator with standard cell culture conditions (a sterile, 5% CO_2_, 95% air, 37°C environment). The controller module was placed outside the incubator but was connected to the flow module using the cable wires with extended length. The 24-h immersion degradation on Mg was repeated for six times, including three times under the flow condition with a flow rate of 0.17 ± 0.06 ml/s for 24 h and three times under the static condition without flow for 24 h. The static condition was achieved by turning off the impedance pump, and the rest of conditions are the same as the flow condition.

### Measurement of the flow rate

The flow rate was determined by measuring the frequency of water droplets from the non-immersed outlet tube into the medium reservoir. The number of droplets *N* among a period of *t*, and the average volume of each droplet *V* were measured to calculate the flow rate *R* based on Equation (1) below.

(1) $$R=\frac{NV}{t}$$

The average volume of each droplet *V* was calculated from the outline of droplet captured by an optical video. An assumption was made that the droplet was considered an ellipsoid. The calculation used the following Equation (2):

(2) $$V=\frac{4\pi (abc)}{3}$$

The a, b and c are the half axes of the assumed ellipsoid. In this study, the two half axes in the horizontal direction (*b* and *c*) were approximated to be the same length. The volumes of six droplets were calculated to obtain the average volume of the droplet. For each run of the flow device, about 10 s of flow were recorded to calculate the frequency of droplets. The experiments were repeated three times and the data were analysed based on the triplicate experiments under flow versus static conditions for Mg samples.

The flow rate was measured a few seconds after the system was turned on to make sure the flow was stable. For all groups, the stable dripping of media from the outlet tube was observed immediately after the system was turned on, and a video is provided in the Supplementary Material. This method for determining the flow rate was used because it is simple and low cost when compared with other methods such as infrared-based flow meters or turbine-based flow meters that can provide accurate measurements for flow rates, but also increase the cost significantly. Turbine-based flow meters also cause the concerns of media contamination and difficulties in cleaning and sterilization after each cycle of experiments because of direct contact with media.

### Measurement of Mg degradation under flow and static conditions

The mass of Mg samples was measured before and after the immersion degradation to calculate their mass change. The mass change was defined as the mass after 24-h immersion ($M_{\text{f}}$) minus the mass before immersion ($M_{0}$) per surface area (*A*), following the equation (*M*_f_–*M*_0_)/*A.* The mass was measured using an analytical balance (NewClassic MF, MS104S). The thickness of the Mg samples was measured using the line scan mode in a 3D laser scanning microscope (VK-X150, Keyence). The thickness change was defined as the thickness after 24-h immersion ($t_{\text{f}}$) minus the thickness before immersion ($t_{0}$), which equals to $t_{\text{f}}-t_{0}$.

Mg^2+^ ion concentrations in the collected media were determined using an inductively coupled plasma optical emission spectrometer (ICP-OES, Optima 8000, PerkinElmer). The ICP-OES was calibrated before the measurement using the standard solutions of Mg^2+^ ions. To minimize the matrix effect, the collected media were diluted at the ratio of 1 : 100.

### Characterization of the degradation products on the surface of Mg samples after 24-h immersion under flow and static conditions

The surface morphology and composition of Mg samples after 24-h immersion degradation under flow and static conditions were characterized using the scanning electron microscope (SEM, Nova NanoSEM 450, FEI Co., Hillsboro, OR, USA) with the attached detector for energy dispersive X-ray spectroscopy (EDS, Nova NanoSEM 450, FEI Co., Hillsboro, OR, USA, X-Max 450). The surface elemental composition and distribution were analysed using the EDS detector and the AZtecEnergy software (Oxford Instruments, Abingdon, Oxfordshire, UK). Elemental mapping combined with SEM was used for investigating the degradation products on Mg samples after 24-h immersion degradation under flow and static conditions while EDS point analysis was used for analysing specific features in the degradation layers. The SEM and EDS analyses were carried out at an accelerating voltage of 20 kV and the SEM images were obtained at an original magnification of 500 x and 5000x. The phases of Mg after 24-h immersion study under flow and static condition were analysed using X-ray diffraction (XRD; Empyrean, PANalytical) at 45 KV and 40 mA with 2*θ* angles from 10° to 80° at a step size of 0.002°. The diffraction peaks were identified based on the international center for diffraction data database using HighScore software (PANAlytical).

## Results

Mg degrades by reacting with water in physiological environment, and releasing Mg^2+^ and OH^−^ ions, as reported previously [18, 20]. The ions, proteins and cells in the *in vitro* culture could affect Mg degradation processes and formation of degradation layers with precipitates from media. The effects of ions and proteins on Mg degradation and mechanisms have been reported and discussed previously [21, 22].

### The change of mass and thickness of Mg samples under flow versus static conditions

**Figure 2:**
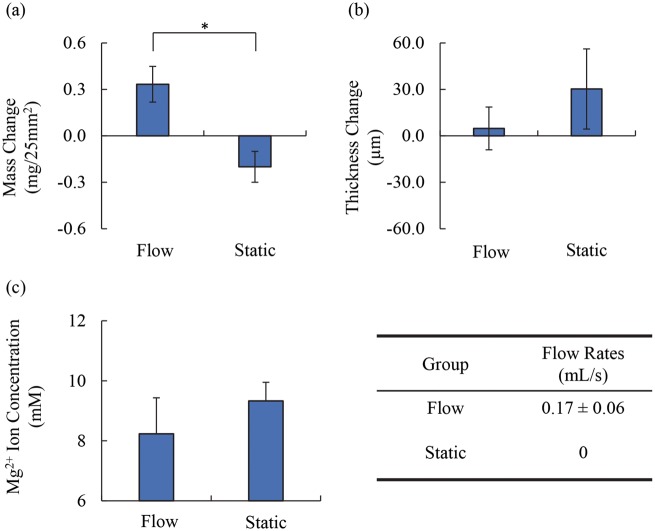
Comparison of Mg degradation under flow versus static conditions. The change of (**a**) mass and (**b**) thickness after the immersion test. (**c**) Mg^2+^ ion concentrations in the culture media after the immersion test under flow versus static condition. The table shows the estimated flow rates by counting the water droplets based on a video. The video is provided in Supplementary Material. Values are average ± SD. *p < 0.05

Figure 2a shows the mass change of Mg. Mg under flow condition showed mass increase while Mg under static condition showed mass loss; the mass change under flow was statistically greater than that under static condition. Figure 2b shows the thickness change of Mg under the flow condition did not statistically differ from the Mg under the static condition, but the thickness of Mg in average was less under the flow condition than Mg under the static condition.

### The Mg^2+^ ion concentration in the media after Mg degradation under flow versus static conditions

Figure 2c shows the Mg^2+^ ion concentrations in the media collected after 24-h immersion degradation. The media for Mg under flow condition was not significantly different from the media for Mg at static condition, but the flow condition shows lower average Mg^2+^ ion concentration.

### The flow rates of media in the flow condition

The table in Fig. 2 shows the flow rate of the media in the flow loop. The flow rate was 0.17 ± 0.06 ml/s at a pressing condition of impedance pump with a cycle of 45−µs press and 100−µs release. The flow rate was 0 for the static condition.

### Degradation products on the surface of Mg samples under flow versus static condition

**Figure 3:**
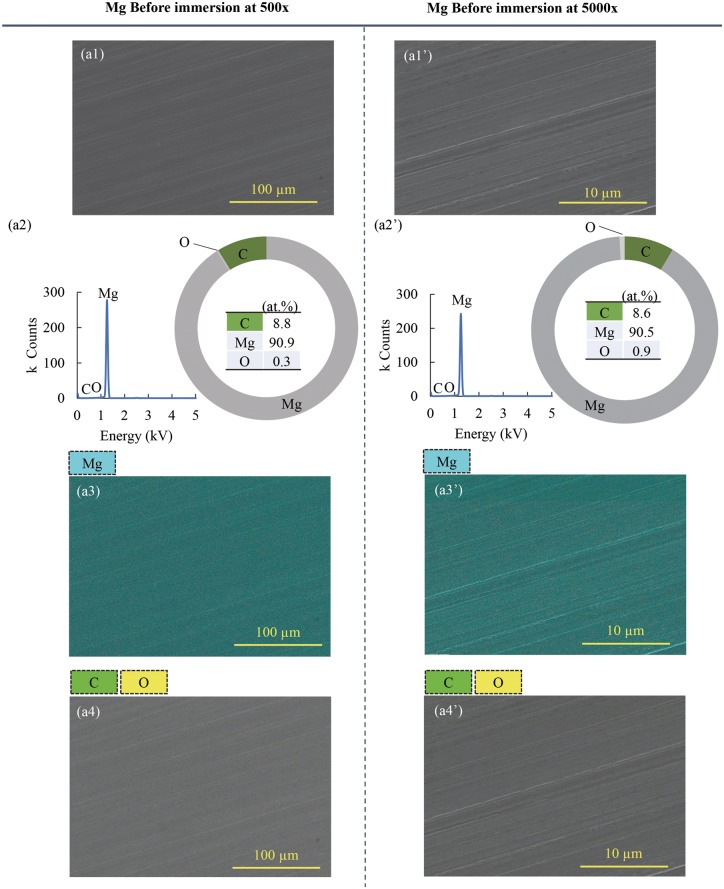
Surface morphology and elemental compositions of Mg before the immersion test. (**a1**) SEM image of the surface of Mg before immersion at an original magnification of 500x and (**a1’**) SEM image of the surface of Mg before immersion at an original magnification of 5000x. (**a2**, **a2’**) show the elemental compositions and EDS spectra of the entire area of the SEM images of (a1) or (a1’), respectively. (**a3**, **a3’**) EDS elemental distribution maps of Mg on the surface of Mg tested. (**a4**, **a4’**) EDS elemental distribution maps of C and O on the surface of Mg tested. The scale bars for (a1, a3, a4) are 100 µm and for (a1’, a3’, a4’) are 10 µm

Figure 3a1 and a1’ shows the surface microstructures of the Mg samples before immersion at an original magnification of 500x and 5000x, respectively. The surfaces appeared homogenous and clean, and some polishing trace was observed at the high magnification (Fig. 3a1’). Figure 3a2 and a2’ show the elemental composition of the area corresponding to the imaging area in Figs. 3a1 and a1’, respectively. The surfaces of Mg samples showed similar compositions at both low and high magnifications; specifically, 90.9 at % Mg and 0.3 at % O in Fig. 3a2 and 90.5 at % Mg and 0.9 at % O in Fig. 3a2’. Figure 3a3 and a3’ show the EDS elemental mapping of Mg on the area corresponding to the imaging area in Figs. 3a1 and a1’, respectively. Figures 3a4 and a4’ show the EDS elemental mapping of carbon (C) and oxygen (O) on the area corresponding to the imaging area in Figs. 3a1 and a1’, respectively.

**Figure 4:**
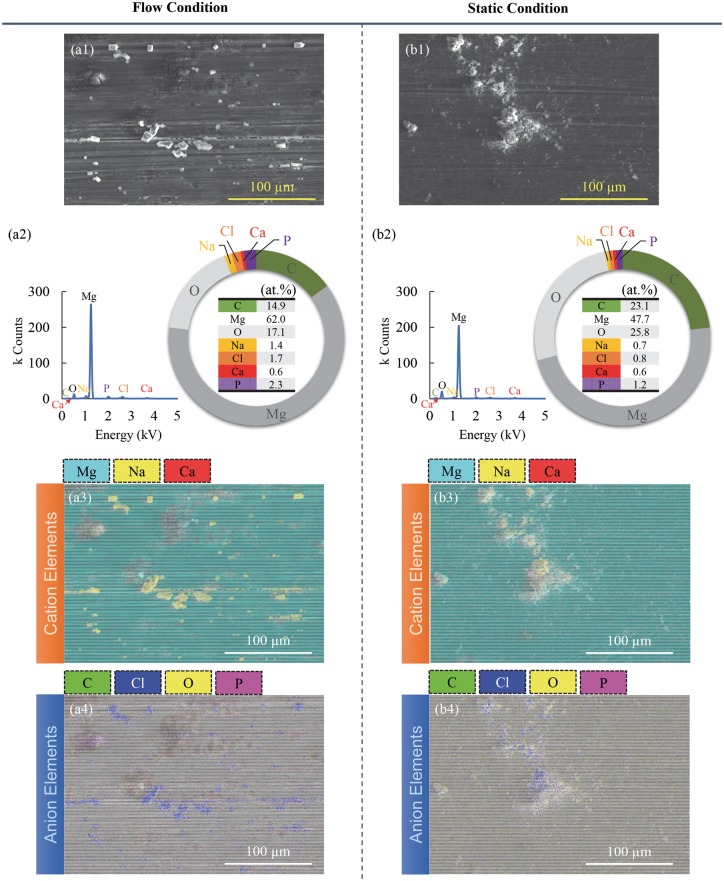
Surface morphology and elemental compositions of Mg after the immersion test under flow versus static conditions. (**a1**, **b1**) show the SEM images of the surface of Mg after the immersion test under (a1) flow and (b1) static conditions. (**a2**, **b2**) show the elemental compositions and EDS spectra of the entire area of the SEM images of (a1) or (b1), respectively. (**a3**, **b3**) show the EDS elemental distribution maps of cation elements (Mg, Na, Ca) on the surface of Mg tested under (a3) flow versus (b3) static conditions. The EDS elemental distribution maps of anion elements (C, Cl, O, P) on the surface of Mg tested under (**a4**) flow versus (**b4**) static conditions. The original magnification for (a1), (b1), (a3), (b3), (a4) and (b4) is 500x. The scale bars for (a1), (b1), (a3), (b3), (a4) and (b4) are 100 µm

Figure 4a1 shows the surface microstructures of the Mg samples tested under flow condition. Some cubic degradation products can be explicitly spotted on the SEM image in Fig. 4a1. Figure 4a2 shows the elemental composition of the area corresponding to the area shown in Fig. 4a1 and the corresponding EDS spectra. The surface mostly contained Mg (62.0 at.%), O (17.1 at.%) and C (14.9 at.%). The full list of elemental composition is shown in Fig. 4a2. Figures 4a3 and a4 shows the EDS elemental mapping on the surface of Mg tested under flow condition. To be visually explicit, we separately presented the EDS mapping of the cation elements (Fig. 4a3) and the EDS mapping of the anion elements (Fig. 4a4). The cubic precipitates on the surface appeared to contain mostly Na and Cl elements.

Figure 4b1 shows the surface microstructures of the Mg samples tested at static condition. Precipitates with irregular shapes can be explicitly spotted on the SEM image in Fig. 4b1. Figure 4b2 shows the elemental composition of the area corresponding to the area shown in Fig. 4b1 and the corresponding EDS spectra. The surface mostly contained Mg (47.7 at.%), O (25.8 at.%) and C (23.1 at.%). The full list of element composition is shown in Fig. 4b2. Figures 4b3 and 4b4 shows the EDS elemental mapping of the cation elements (Fig. 4b3) and the anion elements (Fig. 4b4) on the surface of Mg tested under static condition, respectively. The precipitates on the surface appeared to contain mostly Na and Cl elements.

When comparing the elemental composition on the surface, it appears that surface of Mg tested under flow condition contained higher atomic percentage of Mg, Na, Cl, P and lower atomic percentage of C and O. The atomic percentages of Ca element on the surface of Mg tested under flow and static conditions were almost the same.

**Figure 5:**
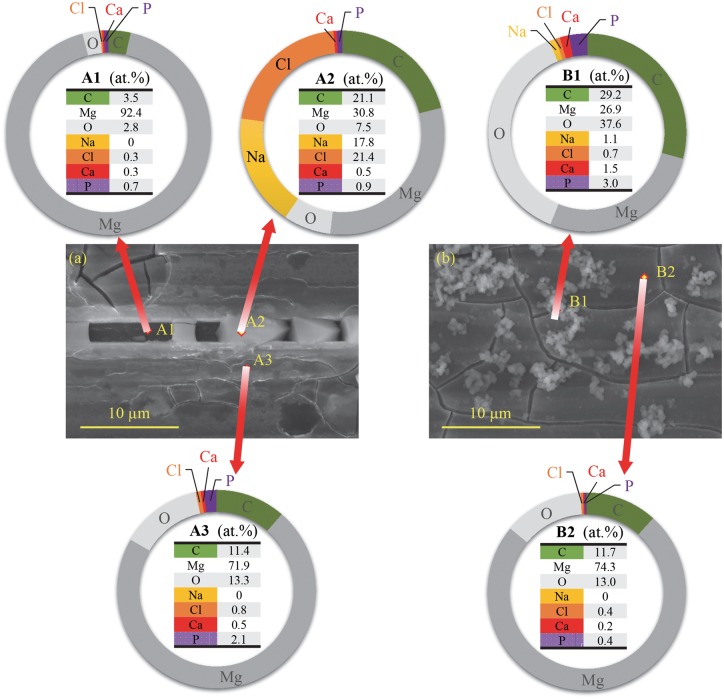
Surface morphology at the original magnification of 5000x and the elemental compositions of different regions on the surface of Mg after the immersion test under flow versus static conditions. (**a**, **b**) The SEM images of the surface after the immersion test under (a) flow versus (b) static conditions. In (a), the elemental compositions of the A1, A2 and A3 points are shown in the respective circular graphs. In (b), the elemental compositions of the B1 and B2 points are shown in the respective circular graphs. The original magnification for (a) and (b) is 5000x. The scale bars for (a) and (b) are 10 µm

Figure 5a shows the microstructures of the cubic precipitates identified on the surface of Mg tested under flow condition at high magnification and the elemental composition at point A1, A2 and A3 as shown in Fig. 5a. The cubic precipitates presented crystal-like morphology. The point A2 showed apparently more abundant Na and Cl elements than point A1 and A3. The point A1 and A3 showed very minimal Na and Cl elements but more Mg content. Point A1 showed the highest Mg and lowest O atomic percentage among the three points.

Figure 5b shows the microstructures of the precipitates identified on the surface of Mg tested under static condition at high magnification and the elemental composition at point B1 and B2 as shown in Fig. 5b. Point B1 showed much lower Mg and much higher C and O atomic percentage than point B2.

**Figure 6:**
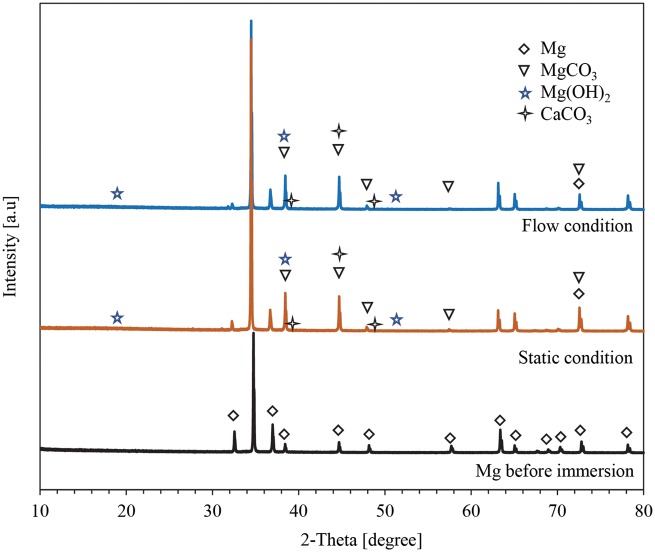
XRD patterns of Mg after the 24-h immersion test under flow versus static condition, as well as Mg before the 24-h immersion test. Phases were identified based on Mg (ICSD pattern 01-071-3765), MgCO_3_ (ICSD pattern 01-070-8513), Mg(OH)_2_ (ICSD pattern 01-074-2580) and CaCO_3_ (ICSD pattern 00-058-0471)

Figure 6 showed the XRD spectra of the Mg after 24-h immersion test under flow condition and static condition. The peaks of XRD spectra for the Mg after 24-h immersion test under flow condition and static condition were similar, but the intensity for the Mg peak at 34.4° is higher under static condition than the flow condition. The XRD spectra of Mg under both flow condition and static condition confirmed the presence of Mg, MgCO_3_, Mg(OH)_2_ and CaCO_3_, which agreed with the EDS analyses that showed the presence of Mg, C, O, Ca. Although the peaks for Mg(OH)_2_ and CaCO_3_ were too small to be clearly visible, the phases were still detected by using the HighScore software. The absence of Na, Cl and P can be ascribed to their low quantity that precipitated on the Mg surface after 24-h immersion test.

## Discussion

### Flow rate of media is adjustable in the flow device

The flow device achieved a flow rate of 0.17 ml/s when the impedance pump was set at a pressing condition of 45−µs press and 100−µs release (Fig. 2). The flow rate that this device can achieve is suitable for mimicking the blood flow rate for vascular applications. The flow rate (0.17 ml/s, Fig. 2) is in the range of the flow rate of blood flow in artery (0.05 ∼ 0.43 ml/s) [23] and is higher than vein (0.02 ∼ 0.08 ml/s) [23]. For coronary artery, the flow rate (∼2.35 ml/s) is generally higher [24]. For brain aneurysm, the flow rate (∼0.03 ml/s) is generally lower [25]. The flow rates can be adjusted by changing the pressing condition of the impedance pump, the position of pressing site relative to the elastic tube and the ID of the flow loop.

### Mg degradation under flow versus static conditions

In this study, Mg under flow condition underwent an increase of mass after the 24-h immersion (Fig. 2a). The increase of the mass (Fig. 2a) was most likely caused by the precipitates on the surface of Mg from the media, which was identified using SEM, EDS and XRD (Figs. 4, 5 and 6). Before immersion, there were no precipitates on the samples (Fig. 3) and the Mg surface contained only Mg phase (Fig. 6) and a small amount of C and a trace amount of O (Fig. 3). The flow condition was maintained continuously at a flow rate of 0.17 ml/s for 24 h in order to examine its capability for cell culture studies that typically require a continuous incubation for 24 h to 72 h. This flow device is stable for longer immersion studies that may last weeks to months, which only involves simple replacement of fresh media at each scheduled time point. It is not conclusive whether this flow condition of 0.17 ml/s increased or decreased the degradation rate of Mg in the short duration of 24 h. The lower average Mg^2+^ ion concentrations in the media for Mg under flow versus static conditions lacked statistical significance (Fig. 2c). Thus, long-term culture studies are needed in the future to fully elucidate the effects of flow on Mg degradation.

The increase in mass (Fig. 2a) and the decrease in Mg^2+^ ion concentration (Fig. 2c) for Mg samples under flow condition indicated a greater degree of precipitation in degradation products than that of static condition. However, the thickness of Mg samples under flow condition did not increase as much as static condition (Fig. 2b). It is speculated that more densely-packed precipitates formed in the degradation layer on Mg surface under flow condition, while more loose and porous degradation layer formed under static condition. Moreover, it is likely that degradation products were not homogeneously distributed on the Mg surface, as shown in the relatively high standard deviations (SD) in the changes of sample thickness under both conditions (Fig. 2b). Interestingly, the deviation of sample thickness under flow condition was less than that under static condition (Fig. 2b), possibly because the degradation layer was more densely packed under flow condition. Although this speculation can account for the differences in sample thickness under different conditions, it must be noted that no statistically significant difference was detected when comparing the sample thickness change under flow versus static conditions.

### Degradation products on the surface of Mg under flow versus static conditions

The degradation products on the surface of Mg presented different morphologies under the flow versus static conditions. The flowing media could have accelerated the crystallization rate of the precipitates, as the precipitates under the flow condition showed crystalline polygonal morphology (Fig. 5a) in contrast to the morphology of precipitates under static condition (Fig. 5b). The flow condition also resulted in the larger size of precipitates than that under the static condition (Fig. 5).

The chemical composition of the precipitates under the flow condition was mostly MgCO_3_. NaCl also precipitated on the Mg surface after 24-h immersion test because the presence of Na and Cl and their atomic ratio of Na to Cl was close to 1 (17.8 at.% for Na and 21.4 % for Cl) (Fig. 4a). In contrast, the precipitates under the static condition could contain less NaCl and more MgCO_3_ because less Na and Cl contents and more C and O contents were detected by EDS (Fig. 4). A small amount of phosphorus (P) was also detected on Mg surface after 24-h immersion test under flow and static conditions because EDS detected P element on both groups of sample.

The flow condition resulted in the formation of a degradation layer that contained MgCO_3_, Mg(OH)_2_ and CaCO_3_ and showed less cracks (Figs. 5 and 6). The chemical composition of the Mg surface under the flow condition (point A3 in Fig. 5a) was similar to that of the Mg surface under static condition (point B2 in Fig. 5b). It is possible that the flow condition might reduce the formation of the cracks and reduce Mg degradation in the first 24 h by spreading the degradation products evenly on the surface.

## Conclusion

A portable flow device driven by the impedance pump was built and tested to be effective for studying the effects of fluidic flow on the properties of biomaterials or devices (e.g. stents) under standard cell culture conditions inside cell incubators. The flow rate in the range of human blood flow rate was achieved, and the flow rates can be easily adjusted for different studies without opening incubator doors. This study proved the concept for this impedance pump driven flow device through the immersion degradation studies on pure Mg under flow versus static conditions, and confirmed this small device was portable for easy placement inside a standard cell culture incubator. The change of mass and thickness, as well as degradation products on the Mg surface, were analysed and compared under flow versus static conditions. Overall, this flow device provides a useful tool that is easy to set up inside a cell incubator to study the effects of various fluidic flow (e.g. blood or urine flow) on the degradation, mechanical and biological properties of novel biodegradable materials and devices (e.g. cardiovascular stents and ureteral stents). This flow device can be used to study *in vitro* cell-material and cell-device interactions inside a cell incubator in the future studies or used to grow cells and tissues under flow in the future for tissue engineering and regenerative medicine applications, considering that the device has easy portals for fast media exchange and the device fits perfectly inside a standard incubator.

## Supplementary Material

Supplementary Data
